# Polyphenols from Foxtail Millet Improve Non-Alcoholic Fatty Liver Disease by Regulating Intestinal Microbiome in Mice

**DOI:** 10.3390/foods13111683

**Published:** 2024-05-27

**Authors:** Israr Ghani, Yuxuan An, Qinqin Qiao, Shuiling He, Zhuoyu Li

**Affiliations:** Key Laboratory of Chemical Biology and Molecular Engineering of National Ministry of Education, Institute of Biotechnology, Shanxi University, Taiyuan 030006, China; 202213001009@email.sxu.edu.cn (I.G.); anyuxuan.com@163.com (Y.A.); qqq13834644457@163.com (Q.Q.); heshuiling2021@163.com (S.H.)

**Keywords:** BPIS, gut microbiome, foxtail millet, NAFLD

## Abstract

Non-alcoholic fatty liver disease (NAFLD) is the most common chronic hepatic manifestation of metabolic dysfunction for which effective interventions are lacking. The burden of NAFLD is increasing at an alarming rate. NAFLD is frequently associated with morbidities such as dyslipidemia, type 2 diabetes mellitus and obesity, etc. The current study explored the potential role of **bound polyphenols from foxtail millet (BPIS)** in treating mice with NAFLD induced by a high-fat diet (HFD). The results indicated the critical role of BPIS in treating NAFLD by effectively restoring the gut microbiota in C57BL/6 mice that received a high-fat diet (HFD) for 12 weeks. At the same time, 16S rRNA analysis demonstrated that BPIS remodeled the overall structure of the gut microbiota from fatty liver diseases towards that of normal counterparts, including ten phylum and twenty genus levels. Further study found that the expression of tight junction proteins was upregulated in the BPIS-treated group. This study provides new insights into the potential NAFLD protective effects induced by polyphenols of foxtail millet.

## 1. Introduction

Non-alcoholic fatty liver disease (NAFLD) has become rapidly increasingly prevalent worldwide. It affects approximately 25–30% of individuals globally, and the prevalence and incidence of NAFLD have increased significantly [1,2,3]. NAFLD is categorized by excessive accumulation of hepatic triglycerides and incorporates a range of disease states, from steatosis to non-alcoholic steatohepatitis, distinguished by the presence of lobular inflammation and hepatocyte ballooning with increasing fibrosis stage, to cirrhosis and hepatocellular carcinoma [4]. Currently, one FDA-approved drug named Rezdiffra (resmetirom) for adult patients with liver scarring due to fatty liver disease has side effects [5]. Further studies are required for novel approaches to NAFLD prevention and treatment. NAFLD is closely related to demographic differences (aging of society), an unnatural lifestyle (overeating, lack of exercise), gut dysbiosis, and cardiovascular–metabolic diseases. Obesity is a key factor in NAFLD and is significantly associated with other NAFLD risk factors [6]. Mice lacking gut microbiota are resistant to diet-induced obesity and hepatic steatosis, and germ-free mice that receive fecal microbiota transplantation from donor mice with metabolic syndrome develop liver steatosis independent of dietary intake, demonstrating that NAFLD occurrence may be associated with the gut microbiome [7,8].

Polyphenols are plants’ secondary metabolites [9]. Many natural compounds are abundant in polyphenols, such as green tea, coffee, cocoa, and plant-sourced foods (vegetables, grains, and fruits), and have positive effects on health (anticancer, hypoglycemic, and anti-inflammatory) [10,11]. There have been studies showing that polyphenol exerts favorable effects on NAFLD, with positive outcomes related to insulin resistance [12], liver fat accumulation, oxidative stress, proinflammatory status, and mitochondrial dysfunction [13,14]. Foxtail millet is extensively grown globally. Foxtail millet bran is the hard outer sheet of foxtail millet, and as a by-product of milling processing, it is often used for livestock and poultry feed [15]. Foxtail millet bran is reported to be rich in vitamins, minerals, essential amino acids, and, especially, polyphenols [16].

Several studies explored BPIS (bound polyphenols of the inner shell) for the treatment of different diseases and reducing the risk of inflammatory bowel diseases and human colorectal cancer through the regulating intestinal microbiome [17,18].

The gut microbiome, a diverse microbial community comprising trillions of bacteria, fungi, viruses, archaea, and protists that encode several orders of magnitude more functional genes than the human genome, can modulate human health and will likely be an integral component of personalized medicine [19]. The diverse microbes that colonize the gut, which are collectively known as the gut microbiota, provide key health benefits. One of the key benefits of colonization is the ability to restrict colonization of the pathogens that trigger different diseases when dysbiosis occurs in the gut microbiota [20]. Under normal circumstances, the relationship between the human host and gut microbiome is mutually beneficial, but perturbations in the gut microbiota have been associated with many chronic diseases [21]. The intestinal epithelial cells and mucus are the first line of defense and limit the translocation of harmful antigens. Possible mechanistic links between the transformed microbiome and fatty liver are emerging and include the potential for bacterial protein to function as ligands for G protein-coupled receptors [22]. Recently, it has become possible to investigate the effects of phenolic compounds on the gut microbiota [23,24]. Many studies suggested that phenolic compounds might affect the gut microbiota to improve hepatic fat deposition, obesity, intestinal inflammation, insulin resistance, oxidative stress, and remodeling of the gut microbiome [25].

The NAFLD preventive effect of BPIS from millet bran is unclear [26] due to the complexity of NAFLD, and the duration, dose, study design, and molecular mechanism involved require further investigation. In this regard, the effects of BPIS on lipid metabolism and inflammation in HF-fed mice were evaluated. Subsequently, we assessed whether BPIS-prevented NAFLD was related to the restoration of the intestinal barrier and gut microbial composition in HF-fed mice. However, BPIS is linked to the alleviation of HF-induced NAFLD through regulating the gut microbiota, and the effect on the liver and colon tissue and intestinal barriers, has not been explained. This study aimed to evaluate the potential role of BPIS in the treatment of NAFLD in mice, with a focus on a possible connection between alterations in lipid metabolism, lipid accumulation effects on the liver and colon tissue, and gut microbiota restoration in NAFLD mice.

## 2. Materials and Methods

### 2.1. Materials

RPMI 1640 medium and fetal bovine serum (FBS) were from Dcell biologics. Standard samples, including foxtail millet, were purchased from Victory Biological Technology Co., Ltd. (Sichuan, China). A PCR kit was purchased from Vanzyme Biotech Co., Ltd. (Beijing, China). HiPerFect Transfection Reagent and EpiTect@Bisulfite Kit (48) were purchased from QIAGEN (Duesseldorf, Germany). Ezup Column Animal Genomic DNA Purification Kit was obtained from Sangon Biotechnology (Shanghai, China). PrimeScript™ RT reagent Kit with gDNA Eraser (Perfect Real Time) and Epi-Taq™ HS (for bisulfite-treated DNA) were purchased from Takara. qPCR SuperMix was obtained from TransGen Biotech (Beijing, China).

### 2.2. Animals

Healthy male C57BL/6N mice (5 weeks old) were purchased from GemPharmatech Co., Ltd. (Nanjing, China). Mice were placed in a specific-pathogen-free (SPF) feeding facility in the Laboratory Animal Center and Animal Laboratory of Nephrology, Shanxi Provincial People’s Hospital (Shanxi, China) at a temperature of 23 ± 2 °C and a humidity of 50 ± 15%, under a 12 h light–dark cycle condition. The health status of the mice was determined via daily observation by technicians under veterinary care. With the permission of the Committee on the Ethics of Animal Experiments of Shanxi University (Shanxi, China) (ethical approval code: SXULL2020046).

Special care was taken to minimize any potential discomfort or distress to the animal during the experimental procedure. All mice were housed in a controlled environment with access to food and water ad libitum. After a week of adaptation, a total of 44 mice with minimal differences were randomly assigned into three groups: For 12 weeks, mice in the normal group (*n* = 11) were fed an ND (270 kcal per 100 g, 10% from fat, 20% from protein, and 70% from carbohydrate; Lab Diet), and the model-making group (*n* = 33) were fed with an HFD (521 kcal per 100 g, 60% from fat, 20% from protein. and 20% from carbohydrate; research diet) for 12 weeks. The BPIS-treated group was given BPIS (100 mg kg^−1^ ) by gavage treatment, which continued for 12 weeks.

### 2.3. Sample Collection

After the 12-week feeding period, the mice were euthanized. Liver, intestine tissue, and feces were quickly frozen and stored at −80 °C for further analysis.

### 2.4. Histopathology and Immunohistochemistry Examination

After 12 weeks of feeding, fresh liver and colon tissue was rapidly immobilized in 4% paraformaldehyde for 24 h, inserted in paraffin wax, and made into 5 μm thick sections. The sections were dewaxed after hydration. Sections of the colon and liver were stained with hematoxylin–eosin (H&E) for histological analysis, Oil Red O (ORO) stain, and immunohistochemistry (IHC) assay. After H&E and IHC staining of the sections, the staining solution was flushed out. After dewatering and sealing, the sections were visualized under a motorized fluorescence microscope (Olympus, Tokyo, Japan), and pictures were saved.

### 2.5. Serum Biomarkers

This service was provided by “Wuhan Servicebio Technology Co., Ltd. (Wuhan, China). Automatic determination by fully automated biochemistry”.

### 2.6. RNA Isolation, cDNA Synthesis, and RT-qPCR

The colons of the mice were comminuted using an automatic freeze grinder (Jing Xin, Shanghai, China). Total RNA was extracted from tissue using Triazole Reagent (Takara, Japan) according to the manufacturer’s protocol. For cDNA synthesis, RNA was reverse-transcribed using the reagent Kit with gDNA Eraser (Takara, Japan), and the concentration of RNA used for the synthesis of cDNA was 500 ng/μL. Primer synthesis was completed by Sangon Biotech (Shanghai, China) as listed in Table 1, and RT-qPCR was performed using Tip Green qPCR SuperMix (TransStart, Beijing, China). Quantification of total RNA was performed by running 1 µL of each sample on Nanodrop. Complementary DNA was prepared by reverse transcription. The qPCR mixture contained 100 ng of tissue cDNA, primer1 (10 µM) 0.4 µL, primer2 (10 µM) 0.4 µL, Template DNA/cDNA × µL, and 2× ChamQ Universal SYBR qPCR Master Mix 10.0 µL, with ddH_2_O to 20.0 µL. PCR amplifications were performed using the following cycling parameters: stage 1 at 95 °C for 30 s; followed by 40 cycles at 95 °C for 10 s, 60 °C for 30 s, and 95 °C for 15 s; 60 °C for 60 s; and 95 °C for 15 s. The cDNA level for each gene was normalized to GAPDH mRNA levels. The primer sequences were as follows:

The copy number was determined from standard curve generation using a synthetic template. Genetic relative quantification was achieved by the 2^−ΔΔCT^ method, utilizing GAPDH as an internal reference. The mRNA levels of genes were analyzed using a real-time PCR thermocycler (Bio-Rad, Hercules, CA, USA) according to the instructions of BlasTaqTM 2× RT-qPCR.

### 2.7. 16S rRNA Gene Sequencing Analysis of Microbiota in the Fecal Contents

16S rRNA gene sequencing was applied to the intestinal microbiome in the fecal contents. Total DNA was isolated by the QIAamp Rapid Fecal DNA Extraction Kit (QIAGEN, Hilden, German). Diluted genomic DNA was used as a template for the amplification of the V3–V4 hypervariable region using specific primers with barcodes. PCR was performed using Phusion^®^ High-Fidelity PCR Master Mix with GC Buffer (New England Biolabs, Salisbury, UK). After the libraries were qualified, the libraries were sequenced using NovaSeq6000. The 16S rRNA sequencing data of the intestinal microbiome were analyzed using QIIME2 software. Venn diagrams were created with the R (Version 3.5.3) Venn Diagram package. Species abundance clustering heatmaps were created with the R (Version 3.1.0) heatmap package. NMDS analysis used the vegan package in R (Version 3.5.3). Differential species analysis was performed with LEfSe software (Version 1.0). Spearman correlation analysis was performed with the R (Version 2.15.3) psych package and the heatmap package.

### 2.8. Statistical Analysis

The mean ± SEM was used to represent the data. One-way ANOVA and Student’s *t*-test analyses were carried out with GraphPad Prism (Version 9.2). Student’s *t*-test was used for single-variable comparisons. Comparisons of means of ≥3 groups were performed by analysis of variance (ANOVA), followed by Tukey’s post hoc test. The data are represented as the mean ± standard deviation (±SD) from at least three independent experiments; *p* values less than 0.05 and 0.01 were considered significantly and highly significantly different compared with the control.

## 3. Results

### 3.1. BPIS Attenuates NAFLD and Reduces Lipid Accumulation

**Figure 1:**
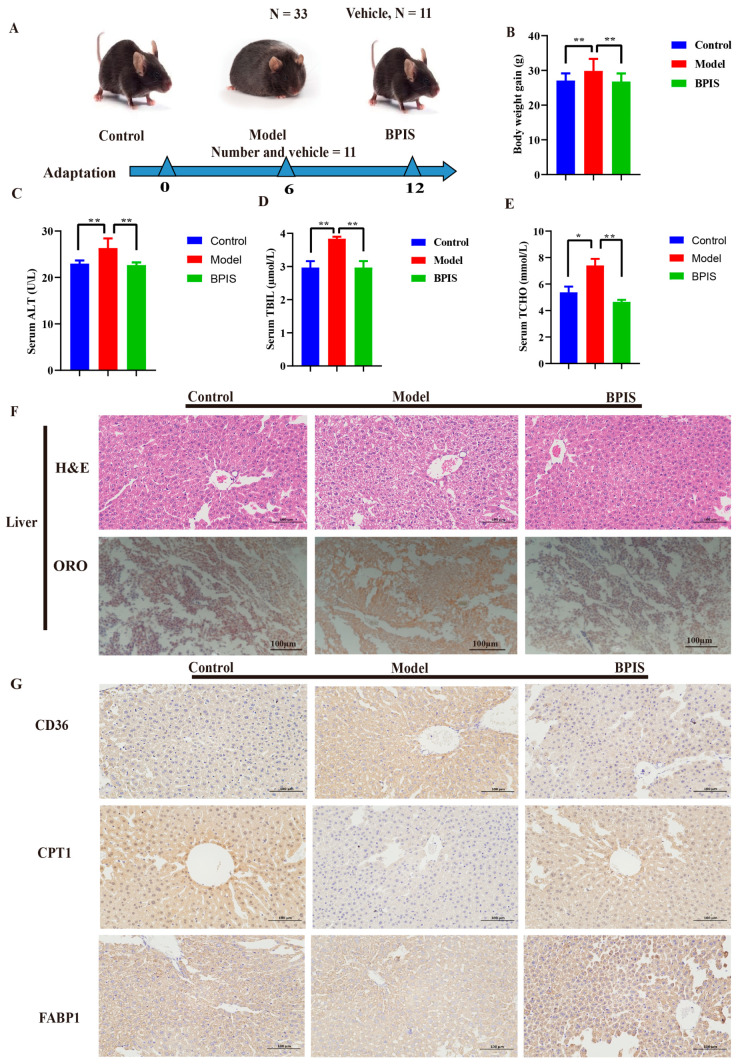
The design of the animal experiment and macroscopic structure of the liver and colon. (**A**) C57BL/6N mice representation. (**B**) Body weight gain. (**C**) Serum alanine aminotransferase (ALT). (**D**) Serum total bilirubin (TBIL). (**E**) Serum total cholesterol (TCHO). (**F**) Images of the H&E-stained and Oil Red O-stained liver. (**G**) IHC-stained liver tissue (significant difference). Data represented as means ± SD (*n* = 3); * *p* < 0.05, ** *p* < 0.01.

To investigate the effect of BPIS on the progression of NAFLD, C57BL/6N mice were grouped and orally treated with BPIS (100 mg kg^−1^) for 12 weeks. The normal-chow-diet-fed C57BL/6N mice were used as a control. The design of the animal experiment is displayed in Figure 1A. The food intake of the control mice was observably greater than that of the other mice, which consumed 30% HFD. The body weight of the BPIS-treated mice was reduced to a normal weight compared to the control group (Figure 1B). HFD ingestion for 12 weeks significantly induced increases in total bilirubin (TBIL), alanine aminotransferase (ALT), and total cholesterol level (TCHO) in the mice (Figure 1C–E).

Next, histopathological observation of hematoxylin and eosin (H&E), Oil Red O stain (ORO-stain) (Figure 1F), and immunohistochemistry (IHC) of the liver tissue (Figure 1G) was performed. There were abundant red lipid vacuoles in the hepatic tissue in the HFD group, unlike in the control group. Co-consumption of an HFD together with BPIS prevented hepatic injury in the mice, and the ORO mean density decreased, indicating that hepatic steatosis was enhanced in H&E-stained liver tissue, showing a near-normal appearance with little cytoplasmic vacuolation, legible cell boundaries, a clear nucleolus, and a detectable nucleus (Figure 1F).

IHC staining was performed for the functional markers for fatty acid translocase (CD36), also known as fatty acid transporter, involved in the pathogenesis of NAFLD. With the BPIS treatment in the HFD mice, the contents of fat were reduced in the liver tissue, with an appearance similar to that of the control group of mice (Figure 1G). CPT1 transports fatty acids from the cytosol to mitochondria, and in that way catalyzes the rate-limiting step of fatty acid oxidation. The HFD group showed higher lipid contents, while the BPIS group tissue displayed lower lipids with an enhanced tissue appearance. Fatty acid-binding protein 1 (FABP1) is involved in free fatty acid uptake. The samples displayed a significant ameliorating effect on the liver tissue in the BPIS-treated group, with a similar appearance to the control group, as shown in Figure 1G. The model group serum markers presented NAFLD, while the BPIS-treated group presented an ameliorating effect on NAFLD. The above results suggest that BPIS significantly ameliorates fatty liver, preventing HFD-induced NAFLD.

### 3.2. Protection of Intestinal Epithelium by BPIS

**Figure 2:**
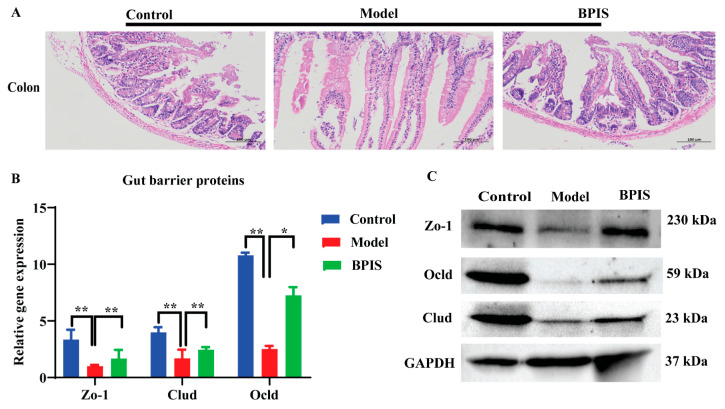
BPIS effect on colon and colonic tight junction proteins (**A**) Images of the H&E-stained colon tissue. (**B**) HFD induces changes in gut permeability, and relative mRNA expression of ZO-1, Claudin, and Occludin. (**C**) Protein abundance of Zo-1, Claudin, and Occludin in the colon, with GAPDH applied as a loading control. Data represented as means ± SD (*n* = 3); * *p* < 0.05, ** *p* < 0.01.

Tight junction proteins, including ZO-1, Occludin, and Claudin, play crucial roles in maintaining gut health and preventing the entry of harmful substances into the body. A leaky gut allows the translocation of bacteria and bacterial products. Dysbiosis can disrupt the intestinal barrier function and contribute to the progression of liver diseases. The data of the histopathological analysis showed that the epithelial architecture of the model group was destroyed in the model group, while after BPIS treatment the colon architecture was completely restored, with a similar appearance to the control group (Figure 2A). The qRT-PCR and Western blot results showed that tight junction proteins were significantly inhibited in HFD mice colon, and decreased levels were observed after BPIS treatment (Figure 2B). The gene expression of Zo-1, Claudin, and Occludin in the mice colon tissue was upregulated after BPIS treatment, and the results were confirmed three times. These results indicate that BPIS treatment improved intestinal barrier dysfunction.

### 3.3. BPIS Regulates the Gut Microbiome in C57BL/6N Mice

**Figure 3:**
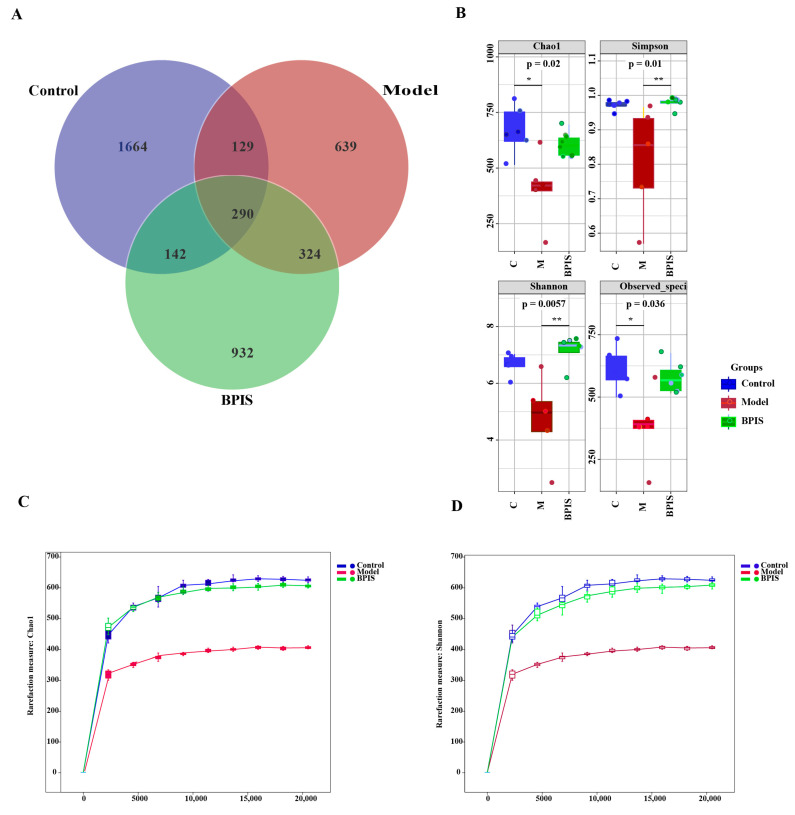
α-diversity and β-diversity analysis of the fecal microbiota communities based on OTUs. (**A**) Venn diagram of different groups. (**B**) Comparison of the diversity indices among different groups: Chao1 index; Simpson; Shannon; observed species. (**C**) Rarefaction curve of Chao1 index. (**D**) Rarefaction curve of Shannon index. Data represented as means ± SD (*n* = 3); * *p* < 0.05, ** *p* < 0.01.

To determine the effect of BPIS on the gut microbiota of NAFLD mice, high-throughput sequencing of 16S rRNA in the cecal content was performed. In this study, the gut microbiota in fecal samples from three groups, control, model, and BPIS, were analyzed for the effect of BPIS on ameliorating NAFLD. According to the results of the OTU cluster analysis, there were 1664 unique OTUs in the control group, 639 in the model group, 932 in the BPIS group, and 290 OTUs shared among the three groups (Figure 3A). The diversity and community richness of the intestinal microbiota were assessed by an alpha diversity analysis using the Chao1 and Shannon indices. A comparable change was found in the value of the Shannon and Simpson indices, indicating that BPIS could effectively inhibit the excessive proliferation of the gut microbiome induced by the chronic consumption of an HFD. A series of analyses were carried out to reveal the critical intestinal microorganisms which BPIS suppressed in the HF-fed mice (Figure 3B) (Chao1 index, *p* < 0.05; Shannon index, *p* < 0.05). The richness and diversity of the intestinal microbiota were evaluated by an α-diversity analysis using Chao1 and observed species (Figure 3B). In addition, the species rarefaction curve (Figure 3C) also presented the same difference in the richness (Chao1) and observed species between the model and other groups. This demonstrated that the current sequencing depth was sufficient to reflect the microbial diversity of the samples. Therefore, BPIS treatment is beneficial for regulating gut microbiota diversity.

### 3.4. BPIS Modulates the Complete Structure of the Gut Microbiome

**Figure 4:**
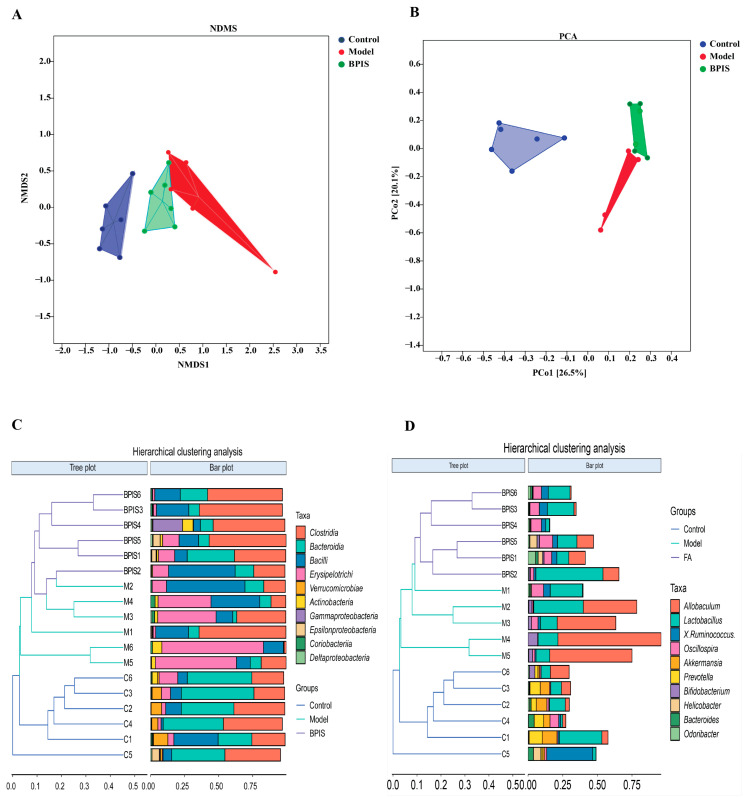
β-diversity analysis of the fecal microbiota communities based on OTUs. (**A**) Principal component analysis (PCA). The percentage variation explained by each principal coordinate is indicated on the axes. (**B**) UniFrac distance-based nonmetric multidimensional scaling (NMDS). (**C**) UniFrac distance-based unweighted pair-group method with arithmetic means (UPGMA) analysis; the shorter the branching length between samples, the more similar the two samples. (**D**) UniFrac distance-based unweighted pair-group method with arithmetic means (UPGMA) analysis.

To investigate the effect of BPIS treatment on the gut microbiome of NAFLD mice, the β-analysis employing several unsupervised multivariate statistical assessments, including PCA, UniFrac NMDS, and UniFrac UPGMA, was performed. As shown in the PCA (Figure 4A) and UniFrac NMDS (Figure 4B), the samples of each group are distinctly clustered. In contrast, the model group indicated low gut community structure compared to the control group, while the gut community structure was recovered by BPIS treatment. UniFrac UPGMA indicated that significant separation appeared between the control, model, and BPIS-treated groups (Figure 4C). These analyses confirmed the effect of BPIS on microbiome structure remodeling in NAFLD mice.

### 3.5. BPIS Regulates the Abundance of Certain Bacteria in Mice

**Figure 5:**
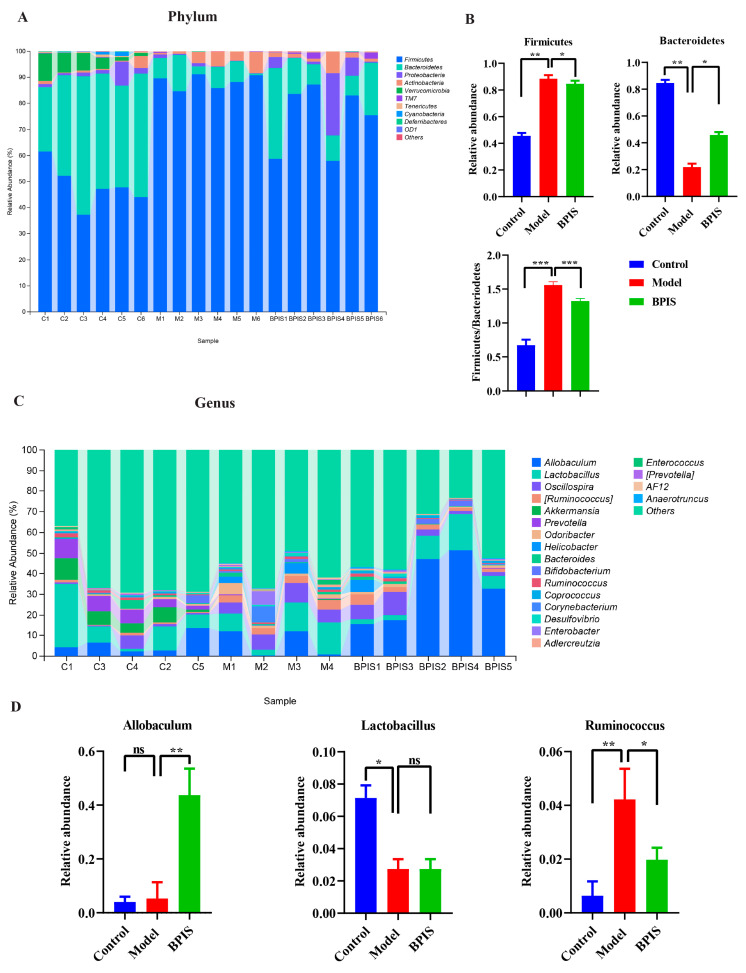
Taxonomy analysis of microbiome components. (**A**) Relative abundance of the top 10 phyla from each sample, compared by Metastatic analysis. (**B**) Significant intergroup differences in two phyla and ratio of Firmicutes to Bacteroidetes. (**C**) Relative abundance of genera, ranking the top 20 from each sample. Significant intergroup differences were found in four genera. (**D**) Significant inter group differences. Data represented as means ± SD (*n* = 4); * *p* < 0.05, ** *p* < 0.01, and *** *p* < 0.001 versus the model groups.

To evaluate the effect of BPIS on the microbiome makeup remodeling, we examined changes in bacterial abundance at the phylum and genus levels by taxon analysis. At the phylum level, a total of ten phyla were shared by all samples, and the most abundant were Firmicutes and Bacteroidetes (Figure 5A,B). BPIS treatment significantly increased the relative abundance of *Firmicutes* (*p* < 0.05), but reduced *Bacteroidetes* (*p* < 0.05) (Figure 5B). Moreover, while the *Firmicutes*/*Bacteroidetes* (F/B) ratio was elevated in the colon of the HF-fed mice, feeding with BPIS effectively inhibited this increase in the F/B ratio. At the genus level, as shown in Figure 5C,D, three genera that positively affected the prevention of NAFLD, including *Allobaculum* (*p* < 0.01) *was* increased with BPIS treatment, *Lactobacillus* (*p* < 0.05) was same prevalence as model, and *Ruminococcus* (*p* < 0.05) were significantly increased as compared to control. Next, the co-occurrence or co-exclusion analysis revealed the correlation between different microbiota compositions. The above data demonstrate that BPIS modulates the overall structure of the gut microbiome by controlling the abundance of certain bacteria. At the genus level, *Allobaculum* was the most abundant recovered genera (Figure 5C,D). The effect of BPIS treatment on the intestinal microbiome composition was mainly explained by the differences in the relative abundance of *Allobaculum*. The mechanism of BPIS improves NAFLD while reversing the gut microbiota, which provides rational support for the use of BPIS as an adjuvant therapy for NAFLD.

## 4. Discussion

Recently, polyphenols have gained the attention of researchers in the prevention and treatment of diseases due to their high availability of bioactive compounds and high biological activities. Plant secondary metabolites can transform intestinal microbial components and produce intestinal metabolites such as methane, hydrogen, and vitamin B complex after microbiome fermentation [27]. NAFLD is highly prevalent worldwide; identifiable risk factors include diabetes type 2, hyperlipidemia, and obesity [28]. The evidence suggests that polyphenols possess promising effects against NAFLD through a variety of molecular mechanisms including the activation of β-oxidation, adipocyte differentiation, and inhibition of free fatty acid uptake and lipogenesis [29]. However, the mechanism by which polyphenols affect NAFLD through the gut microbiome is unclear.

Several studies revealed that the gut microbiota is associated with numerous human diseases. The gut microbiota plays a significant role in the prevention of diseases such as NAFLD and hepatic encephalopathy-associated diseases. Researchers have proved that dysbiosis of the intestinal microbiome is closely associated with lifestyle and leads to the development of NAFLD. The liver can be greatly affected by changes in the gut microbiota due to the entry of gut microbiota metabolites and gut bacteria [30]. The current study shows the medicinal value of BPIS for NAFLD and that it regulates the gut microbiota in mice models. The results displayed a significant ameliorating effect of BPIS on NAFLD. Previous studies reported, at the phylum level, a shift toward an increase in *Firmicutes* and *Bacteroidetes* in obese and high-fructose-fed mice [31] and humans, while weight loss was accompanied by an already lower abundance of Bacteroidetes in obese subjects [32,33]. Currently, there is debate on the validity of the *Bacteroidetes*/*Firmicutes* ratio as a marker of metabolic changes in mice and humans [14,34]. In the present study, *Firmicutes* were increased, while *Bacteroidetes* were decreased compared to the control group after the treatment of BPIS (Figure 5A).

According to a study, Allobaculum was not as well detected in an HFD group of mice; however, when quercetin supplementation was added to the HFD, the relative abundance of these taxa in the NAFLD mice increased dramatically [32], while our results showed that *Allobaculum* increased with BPIS treatment as compared to the control group of mice, and this correlates positively with NAFLD. In the current study, *Allobaculum* increased as compared to control and model group and *Lactobacillus* was same level as model group, while *Ruminococcus* genus was increased as compared to control group with the treatment of BPIS applied to the NAFLD mice as compared to the model group. Other studies reported that *Lactobacillus* genera species administered to C57BL/6j mice significantly improved HFD-related hepatic steatosis and liver damage and increased hepatic expression of peroxisome proliferator-activated receptors and insulin resistance [35,36,37].

NAFLD is studied as the excessive accumulation of fats in hepatocytes. Unlike other prevalent diseases, NAFLD receives little attention, but it is increasing swiftly. The liver is a crucial organ in the human body responsible for an array of functions including metabolism, detoxification, immunity, and vitamin storage. If the liver does not process or break down fats, they will accumulate in hepatocytes. NAFLD is strongly associated with metabolism syndrome and is the cause of chronic liver disease and cirrhosis [38]. The tight junctions of intestinal epithelial cells, as a dynamic permeability barrier, have a double function of preventing potential toxic substances and allowing nutrients to enter the body [39]. In the current study, H&E staining displayed lesions in the liver and colon tissue of mice fed with a high-fat diet, and the colon had tight junction dysfunctions, which are another cause of “leaky gut” which causes inflammatory progression in mice. The tight junction is gathered by the transmembrane such as Occludin, different Claudins, and Zo proteins in the peripheral membrane [40]. However, the liver was repaired after BPIS treatment, and leaky colon tissue was significantly restored (Figure 2). We found that the mRNA and protein expression levels of the tight junction proteins (ZO-1, cloud, and Occludin) in the colon were increased following BPIS administration in BPIS-treated mice, indicating that BPIS could avoid endotoxin accumulation in blood by protecting the gut’s physical barriers. Previous studies reported that the gut microbiota of NAFLD patients reduced the diversity compared to healthy individuals. Under dysbiosis conditions, homeostasis is not capable of being maintained by the gut microbiota, and as a result, disruption of the intestinal barrier occurs [41,42]. A study reported the effect of a synbiotic combination of probiotics and prebiotics improving biochemicals such as triglycerides (TGs), total cholesterol (TC), modification of lipid metabolism, and inflammation by activating the AMPK and NFκB signaling pathways, as well as the intestinal barrier dysfunction and inflammation caused by a high-fat diet [37]. These results are similar to those of our BPIS treatment of the NAFLD mice group.

The current study findings strongly suggest that BPIS can be used to maintain homeostasis of the gut microbiota, serve as a marker in various pathologies, and treat NAFLD. In summary, we found that BPIS treatment mediates increased beneficial bacteria and decreased pathogenic bacteria in the gut microbiota of a fatty liver-diseased mice model and is a key contributor to ameliorating NAFLD.

## 5. Possible Limitations of the Study

While studying the potential role of bound polyphenols from foxtail millet (BPIS) in treating NAFLD in mice induced by a high-fat diet (HFD) offers insightful information, there are some possible limitations to consider. The study duration was 12 weeks, whereas longer-term studies could provide more information on the sustained effects of BPIS treatment on NAFLD development. The number of mice used in the study may impact the generalizability of the findings; greater statistical power and dependability of the results can be achieved with larger sample sizes. Moreover, human clinical trials are required to confirm the safety and effectiveness of BPIS in treating non-alcoholic fatty liver disease (NAFLD). We believe that considering these limitations can help direct future research.

## 6. Conclusions

In conclusion, we investigated that BPIS is an effective agent for reducing lipid buildup in the liver, improving inflammation in fatty liver mice, and enhancing the gut microbiome, providing firm evidence that BPIS treatment significantly mitigated NAFLD in high-fat-diet-induced mice. The specific mechanism by which BPIS interacts with the gut microbiome to exert its beneficial effects may not be fully understood, but BPIS played a significant role in ameliorating NAFLD, and this paves the way for a novel therapeutic agent. It is essential to conduct further research to elucidate the specific pathways and connections involved in the modulation of the intestinal microbiome by BPIS.

## Figures and Tables

**Table 1:** Cldn1, Ocld, Zo1 and GAPDH primers (forward and reverse primer).

Gene	Forward Primer	Reverse Primer
*Cldn1*	TGGCTATGGAGGCGGCTATGG	CCTGAGCGGTCACGATGTTGTC
*Ocld*	TGGCTATGGAGGCGGCTATGG	AAGGAAGCGATGAAGCAGAAGG
*Zo1*	CCACCTCGCACGCATCACAG	TGGTCCTTCACCTCTGAGCACTAC
*GAPDH*	ACCCACTCCTCCACCTTTGA	AAGGAAGCGATGAAGCAGAAGG

## Data Availability

The original contributions presented in the study are included in the article, further inquiries can be directed to the corresponding author.
